# Automated landmark-based symmetric and standard alignment of skull base structures on CT

**DOI:** 10.1016/j.ynirp.2025.100260

**Published:** 2025-04-04

**Authors:** Justin A. Cramer, Trevor Huff, Sean Kelly, Daniel Welch, Devin DeLuna, Conner Beyersdorf, Robin High, Matthew White

**Affiliations:** a Mayo Clinic Arizona, 5711 E Mayo Blvd, Phoenix, AZ, 85054, USA; b University of Arizona, 1501 N. Campbell, Tucson, AZ, 85724, USA; c University of Nebraska Medical Center, 42nd and Emile, Omaha, NE, 68198, USA; d University Hospitals, 11100 Euclid Avenue, Cleveland, OH, 44106, USA

**Keywords:** U-Net, Alignment, Head CT, Landmark, Registration, AC-PC line

## Abstract

**Introduction:**

Symmetry and standard alignment are crucial in both clinical interpretation and research on head CT studies. Registration to a standard template is the traditional method for alignment, yet registration does not guarantee precise alignment of any given structure. This study introduces a method for aligning skull base structures while still achieving a standard anterior commissure-posterior commissure (AC-PC)-like orientation on head CT studies using landmarks, specifically the cochleas and nasal bridge.

**Methods:**

A retrospective study was conducted using head CTs from various General Electric scanners. Landmarks were manually annotated, and a 3D U-Net was trained for landmark identification. Landmark-based alignment was then performed on a test dataset and assessed in two different ways: whole head and skull base alignment. Whole head alignment was assessed quantitatively by expert review. Skull base alignment was then assessed at the cochleas, comparing their alignment between this landmark-based technique and registration to a template.

**Results:**

This landmark-based technique significantly improved whole head and skull base alignment of head CT studies. Whole head alignment reduced average deviations of 5, 11, and 4° in the axial, sagittal, and coronal planes to 1, 5, and 2° respectively. Meanwhile, skull base alignment assessed via the cochlea was also improved relative to traditional registration. For the landmark technique, the cochleas were deviated from perfect by a mean of 0.552 and 0.511 mm along the y and z axes compared to 2.110 and 2.506 mm with registration.

**Conclusion:**

This study demonstrates a simple landmark-based technique for aligning the cochleas on head CT studies while approximating whole head AC-PC orientation, which has applications in both clinical and research settings, particularly for studies focused on the skull base.

## 1 Introduction

Two factors are particularly important for clinical interpretation and research on head imaging: symmetry and standard alignment. A symmetric viewing experience across the sagittal plane makes pathologic asymmetry more conspicuous, and aids analysis of structures for research purposes (George et al., 2023). Beyond just symmetry, alignment of head imaging in a predictable manner in all 3 planes allows for a consistent clinical interpretation experience, and is a well-recognized preprocessing step in brain imaging research (Rorden et al., 2012). The anterior commissure - posterior commissure (AC-PC) line defines the standard axial plane for brain alignment, and is the goal of scanning technologists (Weiss et al., 2004) as well as the basis for brain templates (Rorden et al., 2012). However, routine clinical scanning, particularly with head CTs, commonly falls short of standard alignment (George et al., 2023; Zhang et al., 2019).

Alignment of brain imaging is typically accomplished via registration to a standard template. This is a long-accepted practice for brain MRI, and has more recently been utilized with head CT (Rubbert et al., 2020; Yamin et al., 2020). However, for clinical use and quantitative research, registration must be linear/rigid as opposed to non-linear/deformable, as the physical dimensions of the image must be preserved. Given the inherent asymmetry of cranial structures(Radosevic et al., 2020) and their anatomic variability(Bhattarai et al., 2023), linear registration to a template may produce a robust overall alignment of structures to the AC-PC line, but it cannot guarantee precise alignment/symmetry of any given structure.

Meanwhile, the skull base and face contain numerous discrete anatomic structures that are of both clinical and research interest (Bhattarai et al., 2023; Sepahdari and Mong, 2013; Zhang et al., 2019). Symmetry of these structures is critically important to their reliable quantification, but so is an overall standard and predictable alignment plane. For example, the calculated area of a particular foramen may vary widely with different angulations.

Previous attempts have been made to align skull base structures. One publication describes a method utilizing numerous landmarks to achieve an overall similarity (Chmelik et al., 2019), though without explicit alignment of any given structure. Another one describes alignment of the lateral semicircular canals via convolutional neural network (CNN) segmentation, determining the axial plane of alignment by the angulation of the semicircular canals (Li et al., 2022), which ensures alignment of that particular structure without regard to overall head alignment. To our knowledge, no study has attempted to achieve symmetric alignment of skull base structures while approximating AC-PC head alignment.

Finally, several skull base and facial structures have historically been utilized to guide technologists in scanning. One of these is the orbito-meatal line (OML), previously shown to deviate from the AC-PC line by 9° (Weiss et al., 2004). The cochleas and nasal bridge are two readily identifiable landmarks on head CTs that roughly approximate the OML.

We show that by explicitly identifying the cochleas and nasal bridge with a CNN and aligning them, essentially an automated landmark-based registration, an overall head CT alignment approximating traditional AC-PC alignment can be achieved while producing a more symmetric alignment of skull base structures than achieved with whole brain linear similarity metric registration techniques.

## 2 Materials and methods

Approval was obtained from the University of Nebraska Medical Center Institutional Review Board (# 646-18-EP) for this retrospective study. To summarize, landmark-based head CT alignment was implemented. For this technique, both skull base (cochlear) and whole head alignment was assessed. Whole head alignment was quantified with manual measurements, and skull base alignment was compared with traditional registration.

### 2.1 Landmark selection

The bilateral cochleas and the nasal bridge were selected as landmarks for alignment. These were selected due to their relatively unique appearance and approximation of the OML.

### 2.2 Data gathering

Training data was gathered from noncontrast head CTs collected for a prior study (Huff et al., 2019) (Table 1 – Training Data, and Test Data for Skull Base Alignment). For each subject, a single sequence was collected per exam. These were performed on General Electric (GE, Boston, MA) Revolution and LightSpeed CT scanners at our main hospital and surrounding outpatient clinics. All CTs were obtained as axial/contiguous acquisitions, with 0.625 mm slice thickness and 0.5 mm pixel spacing, reconstructed in GE standard algorithm with iterative reconstruction technique. All CTs were obtained with a 512 x 512 imaging matrix. Imaging was performed with a kV of 120 and smart mA range of 160–475.

**Table 1:** Demographics for training and test data.

Demographics	Training Data	Test Data for Skull Base Alignment	Test Data for Whole Head Alignment
Number of patients	200	96	211
Sex (Male)	104 (52 %)	57 (59 %)	110 (52 %)
Patient age (years)			
Range	7–92	14–91	0.33–92
Mean ( ± SD)	52.8	57	55

Exclusion criteria were excessive motion and significantly deformed or absent landmarks. Additional exclusion criteria of intracranial ventricle distortion for the initial study (Huff et al., 2019) were deemed irrelevant to this study as only bony landmarks were considered.

After training and alignment algorithm implementation (described below), manual validation of alignment accuracy was performed. For this step, additional head CT exams were gathered, one sequence per subject (Table 1 – Test Data for Whole Head Alignment). All CTs were acquired helically, with otherwise identical acquisition parameters to the training dataset described above. This test dataset was acquired helically due to an interval change in institutional head CT protocols. Unique patients were selected. To ensure a mix of unique inpatients and outpatients, all head CTs performed on the first of the month from January through March of 2021 were included. (Consecutive days would excessively exclude repeat inpatients.) Exclusion criteria were excessive motion. The above criteria of deformed or absent landmarks was not specifically screened for in this dataset so as to assess real-world performance.

### 2.3 Training data labeling

Manual labels were placed for each CT image using 3DSlicer (version 4.0.0) (Fedorov et al., 2012). Labels were placed as a 1 cm spherical region of interest (ROI) centered at the cochlear modiolus, and over the nasal bridge centered left-to-right and cranio-caudal at the apex of concavity. Labels were placed by a medical student involved in study conception and landmark selection. 1 cm spherical ROIs were chosen given that predictions would occur at 1/3 the original spatial resolution of the images (128 x 128 downsampled from 512 x 512) due to GPU memory constraints.

### 2.4 U-Net training

The base head CTs and labels were augmented by one additional rotated version of each head CT, with up to a 45° rotation in each plane, doubling the number of head CTs used for training and validation. 90 % were used for training, and 10 % for validation.

**Fig. 1:**
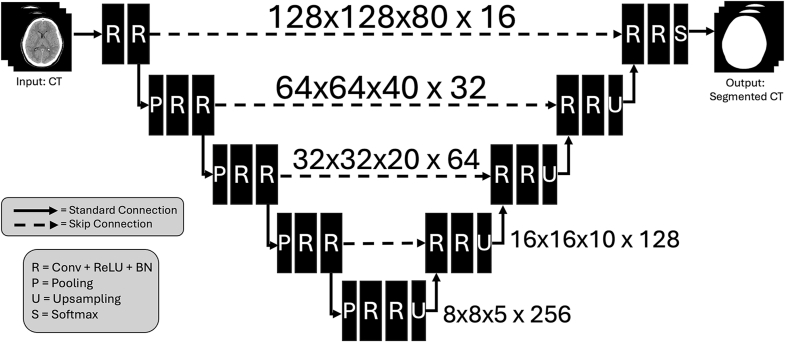
Schematic representation of 3D U-Net utilized for landmark identification. Specifically, 128 × 128 × 80 initial 3D image resolution with 5 channels scaling from 16 to 256 with strides of 2 and 2 residual units per layer (R) are depicted.

A 3D U-Net (Çiçek et al., 2016) was created and trained using the Project MONAI (version 0.9.1) Python toolkit(Consortium, 2024) for the right cochlea, left cochlea, and nasal bridge. A separate model was trained for the right cochlea, left cochlea, and nasal bridge to allow flexibility for additional future landmarks. The U-Net was configured with 5 channels scaling from 16 to 256 with strides of 2 and 2 residual units per layer, summarized in Fig. 1. The training and validation datasets underwent uniform preprocessing to ensure consistency. This included image resampling from 0.5 x 0.5 × 0.625 mm to 1.5 x 1.5 × 1.5 mm due to GPU memory constraints not allowing for full resolution prediction, resulting in image resolution of 128 × 128 × 80. Resampling utilized bilinear interpolation for image data and nearest neighbor for labels. Voxel data was normalized to values between 0 and 1, and orientation was corrected to LPS (Left, Posterior, Superior) coordinates. The network was optimized using the Dice Loss and an Adam optimizer with a learning rate of 0.0001. Training utilized early stopping based on the Dice metric improvement, halting if no progress was observed over 50 epochs, to prevent overfitting. The best-performing model was saved based on the highest observed Dice score during validation. Training reproducibility was ensured through deterministic operations.

### 2.5 Landmark-based registration alignment algorithm implementation

**Fig. 2:**
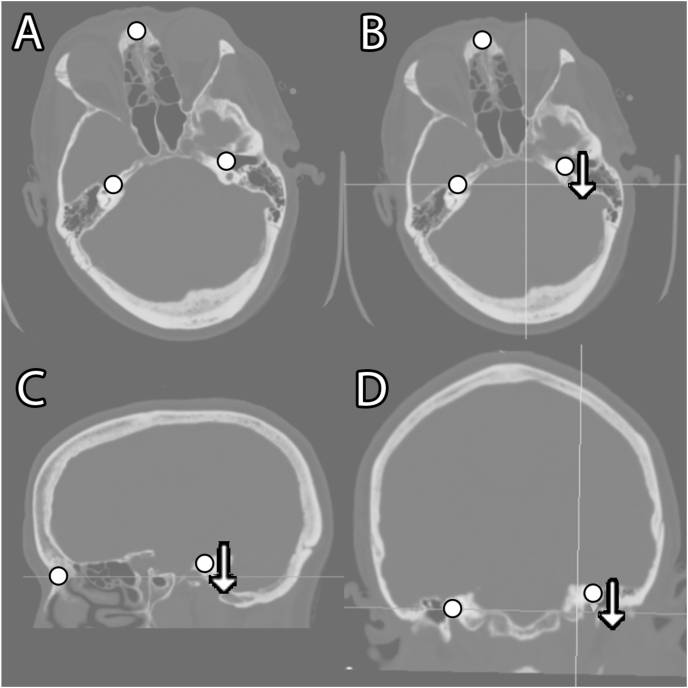
Landmark alignment technique. CT images are shown in a bone window setting to better demonstrate skull base structures. Initial CT (A) is shown in a plane including all three landmarks (black circles on nasal bridge, right cochlea, left cochlea). First, axial rotation to align the cochleas on the y axis (B), then sagittal rotation to align the nasal bridge and right cochlea on the z axis (C), then coronal rotation to align the cochleas on the z axis (D).

Once U-Nets were trained to recognize the cochleas and nasal bridge, the proposed landmark-based registration alignment algorithm was implemented. First, each landmark was identified by the 3D U-Net on a full head CT at a downsampled resolution of 128 × 128 due to GPU memory constraints. The center of mass was calculated for each label using the SciPy (version 1.7.3) center_of_mass method. Then, the head CT was rotated to align the cochleas on the y and z axes and the nasal bridge and right cochlea on the z axis. Fig. 2 summarizes these three rotations that make up the entirety of the alignment: one rotation in each axial/yaw, sagittal/pitch, then coronal/roll planes for three rotations total. These rotations had the effect of placing all three landmarks on the same axial plane, with the cochleas aligned with each other anterior-posterior and cranio-caudal. Rotation was performed as a composite transform using SimpleITK (version 2.0.2) to only incur one interpolation and control the order of rotation(Yaniv et al., 2018).

### 2.6 Landmark-based registration: skull base alignment assessment

Accuracy of alignment for this landmark registration technique was assessed in two ways: whole head and skull base alignment. Skull base alignment was assessed based on cochlear alignment, and accuracy was compared to a more traditional registration technique to further reinforce the point that registration does not reliably align skull base structures. To do this, we used a separate test dataset of head CTs acquired in the same fashion as the training/validation dataset, with identical manually placed labels on the cochleas and nasal bridge. Essentially, we took the labels used for alignment, and instead used them to compare cochlear alignment between two techniques. Each head CT was aligned by the landmark technique as described in section 2.5. Then each head CT was also aligned by registration to the Rorden standard head CT template (Rorden et al., 2012) using ANTsPy (version 0.3.7) with a linear “Similarity” transform (rigid scaling, rotation, and translation) and otherwise default parameters to include mutual information metric. The cochlear labels were transformed in the same manner with both techniques.

Once each head CT was aligned by the two different techniques, accuracy of alignment was assessed based on the transformed cochlea labels in terms of millimeters deviation between right and left cochlea along the y (anterior-posterior) and z (craniocaudal) axes. This was done in regards to the center of mass of each label. Perfect alignment was considered to be y and z deviations of 0. In other words, if the cochleas were aligned perfectly by either technique, then the transformed ground truth cochlea labels should have the same y and z coordinates.

### 2.7 Landmark-based registration: whole head alignment assessment

Whole head alignment accuracy for our landmark registration technique was then assessed manually. The goal was to quantify how close the whole brain alignment was to ideal AC-PC alignment. This was done to reinforce the point that our landmark registration technique not only aligns skull base structures reliably, but also aligns the whole head in a standard fashion.

First, additional head CTs were gathered as described in section 2.2. Of note, these CTs were not manually annotated with landmarks, rather the landmarks were predicted using the trained U-Net. Each head CT was then aligned using the landmark technique. Processing was performed on a computer with an Intel Core i7-9700K CPU (Intel Corporation, Santa Clara, CA), and an NVIDIA GeForce GTX 1080 Ti GPU (Nvidia Corporation, Santa Clara, CA).

**Fig. 3:**
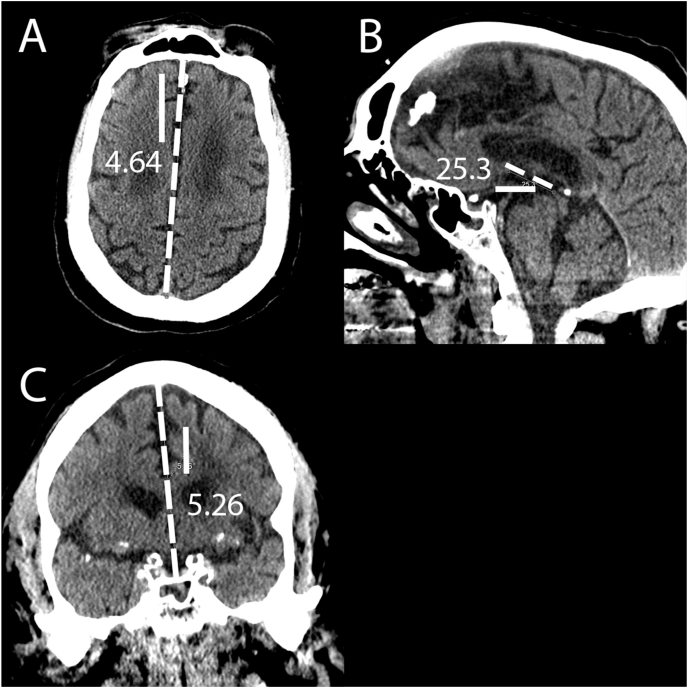
Manual whole head alignment validation measurement technique. CT images are shown in a soft tissue window setting to better demonstrate measurement landmarks on this unaligned head CT. (A) Axial alignment (degrees angulation from the dashed line between the anterior and posterior attachments of the falx) is off by 4.64° in the clockwise direction for a value of +5 (A). (B) Sagittal alignment (degrees angulation from the dashed AC-PC line) is off by 25.3° in the clockwise direction for a value of +25. (C) Coronal alignment (degrees angulation from the dashed line drawn between the center of the sella inferiorly and superior sagittal sinus superiorly) is off by 5.26° in the counter-clockwise direction for a value of −5 (C).

The original CTs along with the aligned versions were sent to a test PACS environment. 5 readers then measured the accuracy of alignment for the original CT and landmark aligned version, with each CT measured by two readers. Readers included an attending neuroradiologist with six years experience, two radiology residents, and two medical students (different than the medical student who performed the labeling). The trainees were educated on the measurement technique via a video walkthrough created by the neuroradiologist. Fig. 3 summarizes the measurement techniques for each plane. Axial alignment was measured as degrees angulation from a line between the anterior and posterior attachments of the falx. Coronal alignment was measured as degrees angulation from a line drawn between the center of the sella and superior sagittal sinus. Sagittal alignment was measured as degrees angulation from the AC-PC line. Misalignment in the clockwise direction was reported as positive degrees, and counter-clockwise misalignment was reported as negative degrees. Degrees were rounded to whole numbers. The average of the two measurements was then utilized.

### 2.8 Statistical analysis

Analysis of the landmark technique whole head alignment accuracy versus original alignment was performed via descriptive statistics utilizing SciPy Python libraries. After a Shapiro-Wilk test determined a non-normal distribution of data, a Wilcoxon signed-rank test of the mean deviations from perfect for each plane was utilized for the paired measurements. Absolute values of the deviations were considered, as the positive and negative values for clockwise and counter-clockwise deviations tended to cancel each other out and artificially minimize differences. Interrater reliability was assessed using the Intraclass Correlation Coefficient (ICC) with Pingouin version 0.5.4 statistical software in Python(Vallat, 2018). Given the random pairing of raters and the averaging of measurements across raters for each subject, the ICC(1,k) model for average measures, absolute agreement, was employed.

Analysis of the landmark technique cochlea alignment accuracy versus similarity metric registration was also performed via descriptive statistics and Wilcoxon signed-rank test of the mean absolute deviations of the cochleas from each other for the same reasons as above, also with the SciPy Python library.

## 3 Results

### 3.1 U-Net training

Training for the right cochlea terminated after 150 epochs with a mean Dice of 0.79, left cochlea after 76 epochs with mean Dice of 0.80, and nasal bridge after 120 epochs with mean Dice of 0.67.

### 3.2 Landmark-based registration: skull base alignment assessment

**Fig. 4:**
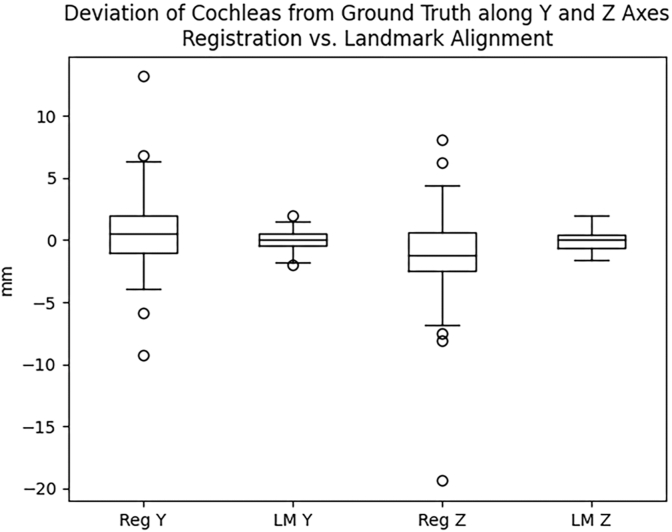
Assessment of Landmark versus Registration Skull Base Alignment. This box plot demonstrates the millimeters (mm) of deviation from perfect symmetric alignment of the cochleas along the y and z axes by original, not absolute values. (Reg = Registration Alignment, LM = Landmark Alignment).

Accuracy of skull base (cochlea) alignment was compared between our landmark registration technique and more traditional similarity metric registration. This was performed on the 96 subject test data set, with each CT containing ground truth labels of the cochleas. Results are summarized in Table 2 and Fig. 4. Similarity metric registration produced a mean deviation between the right and left cochleas of 2.110 and 2.506 mm on the y and z axes respectively, with maximum deviations of 13.184 and 19.375 mm respectively. Visual inspection of outliers demonstrated images very little changed from initial alignment indicating poor overall registration to the template. Landmark method mean deviations were significantly less (p = 0.000), measuring 0.552 and 0.511 mm on the y and z axes respectively, with maximum deviations of 1.953 and 1.937 mm. In other words, our landmark registration method produced a better alignment of the cochleas, more consistently. These differences were statistically significant as assessed by Wilcoxon signed-rank test.

**Table 2:** Landmark skull base alignment results.

Deviation from Perfect Cochlear Alignment (mm)	Registration (mm)	Landmark (mm)	p-value (Wilcoxon)
**Y Deviation**			
Mean	2.110	0.552	0.000
Median	1.465	0.488	
SD	2.050	0.510	
Range	0–13.184	0–1.953	

**Z Deviation**			
Mean	2.506	0.511	0.000
Median	1.875	0.625	
SD	2.473	0.475	
Range	0–19.375	0–1.937

### 3.3 Landmark-based registration: whole head alignment assessment

Subsequently, the same landmark registration technique was assessed in terms of whole head alignment. This was assessed on a 211 subject test dataset via manual measurements as described in the methods.

Landmark alignment failed four times. One failure was on a 4 month-old infant. 2 failures were due to altered anatomy (extensive nasal bridge and other facial bone fractures, prior labyrinthectomy). The fourth was in a patient with marked proptosis where nasal bridge prediction failed.

**Fig. 5:**
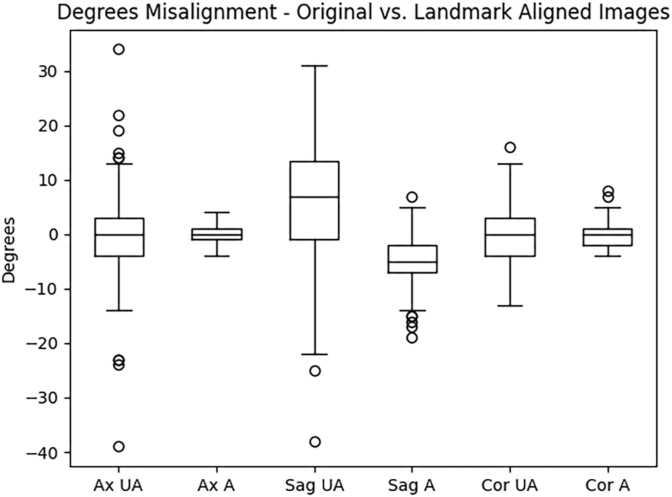
Assessment of Landmark Whole Head Alignment Accuracy. This box plot demonstrates the whole head absolute degrees of malalignment in each plane on the original unaligned acquisition versus the landmark aligned images. (UA = unaligned, A = landmark aligned, Ax = axial, Sag = sagittal, Cor = coronal).

Results are summarized in Table 3 and Fig. 5. The unaligned, original head CTs deviated from perfect by a mean of 5, 11, and 4° in the axial, sagittal, and coronal planes respectively. After alignment, those CTs deviated from perfect alignment by a mean of 1, 5, and 2° respectively. Standard deviation (SD) of deviations in the aligned axial, sagittal, and coronal planes were 1, 4, and 1° respectively, reflecting greater variation in alignment of the sagittal plane.

**Table 3:** Landmark whole head alignment results.

Plane	Degrees Deviation		
	Unaligned	Aligned	p-value (Wilcoxon)
**Axial**			
Mean	5	1	0.000
Median	4	1	
SD	5	1	
Range	0–39	0–4	

**Sagittal**			
Mean	11	5	0.000
Median	9	5	
SD	8	4	
Range	0–38	0–19	

**Coronal**			
Mean	4	2	0.000
Median	3	1	
SD	3	1	
Range	0–16	0–8

When considering average degrees from perfect, not as absolute values, these values were closer to 0 due to averaging together positive (clockwise) and negative (counter-clockwise) values. However, one trend was apparent for the sagittal plane on landmark alignment. The degree deviation was −5, and was 5 as an absolute value. This indicates a consistent average deviation from the “perfect AC-PC” alignment of approximately 5° in the counter-clockwise direction.

Interobserver reliability statistics are reported in Table 4. Measurement of alignment in the axial and coronal planes demonstrated good to excellent reliability, while measurement of sagittal alignment on the landmark aligned images demonstrated moderate reliability (0.62).

**Table 4:** Interrater reliability.

	ICC(1,k)	
Plane	Unaligned (95 % CI)	Landmark Aligned (95 % CI)
Axial	0.99 (0.99–0.99)	0.89 (0.85–0.91)
Coronal	0.98 (0.97–0.98)	0.91 (0.88–0.93)
Sagittal	0.96 (0.95–0.97)	0.62 (0.50–0.71)

## 4 Discussion

This study describes an automated landmark-based registration technique for aligning head CTs based on landmark identification by a U-Net that approximates AC-PC alignment, while also more precisely and consistently aligning skull base structures, specifically the cochleas, when compared to linear similarity metric registration to a template. To our knowledge, this is the first study to assess automated identification and alignment of landmarks which achieve alignment of skull base structures while approximating a standard AC-PC whole brain orientation.

This study also establishes that the cochlea-to-nasal bridge plane deviates from AC-PC alignment by 5° on average, similar to the 9° deviation previously reported for the OML (Weiss et al., 2004). These different values reflect a slightly different plane, as the cochlea is located slightly superior to the external auditory canal/meatus. This is relevant because a 5° correction factor could be applied to our landmark registration technique to more closely approximate AC-PC alignment.

As previously discussed, precise alignment of skull base structures is not guaranteed with linear registration to a standard template. Moreover, simply aligning skull base structures symmetrically across the sagittal plane without regard to axial plane is not sufficient for clinical or research purposes. They must be aligned symmetrically and along a predictable axial plane. For clinical purposes, imaging focused on bony structures, particularly the central skull base such as a temporal bone CT, would particularly benefit from this alignment. For research, imaging subjectively assessing central skull base structures or attempting to manually label them consistently would benefit most from this alignment.

One advantage of this landmark registration technique is relative simplicity and transparency: the cochleas are aligned along the x and z axes, then the cochleas and nasal bridge are aligned along the axial plane via simple rotation transforms. It provides explicit control over alignment based on three bony landmarks. This technique could easily be implemented for CTs with small field-of-views such as CT of the temporal bones, CT of the face, or CT of the sinuses. Registration of such CTs to a whole head standard template may yield less predictable results. Finally, no scaling of structures is required with this method, which could be a component of registration to account for and undo to maintain original physical size.

Identifying these landmarks does allow for alignment via other methods. Registration to a template could be performed first with a final abbreviated transform to align the cochleas without regard to the nasal bridge. This may be more desirable if brain alignment is the primary concern, as perfect AC-PC alignment based on bony landmarks is not possible due to variation between the skull and brain alignment as shown in our data. Alignment of the skull base may be less consistent with this technique. Alternatively, traditional landmark-based registration could be performed based on the identified landmarks, likely with similar results to our method but introducing a component of scaling. Additional landmarks could also be added with relative ease due to the relatively straightforward manual labeling process. For example, a head could be aligned with this landmark alignment technique, with minor axial/y axis rotations and coronal/z axis rotations to more precisely align another specific landmark while still approximately preserving the sagittal/AC-PC orientation.

There are several limitations and considerations to this technique. Importantly, it will not work on infant head CTs who have incompletely ossified skulls. A separately trained model would be required to recognize their landmarks. However, similarity metric registration is similarly limited due to the vastly different appearance of infant skulls. This technique will also not work when the landmarks are severely deformed, such as with extensive facial fractures or congenital malformations of the temporal bone. The algorithm could also fail on patients who are extremely rotated given the 45° rotation augmentation to the training data for this version.

Another limitation is that similarity metric registration to a template essentially failed occasionally and lead to outliers in cochlea alignment. It is possible the similarity metric could have performed better with further fine-tuning of registration parameters, though this also highlights a benefit of our more simple and automated landmark-based technique.

A final limitation is that this landmark alignment method only uses the cochleas and nasal bridge, thus is only guaranteed to precisely align these structures. While structures at least immediately surrounding the cochlea are likely more precisely aligned with this landmark technique, this was not specifically evaluated and is a direction for future investigation. Moreover, training of additional landmarks may be necessary to precisely align a specific structure by the method described above.

For longitudinal follow-up, this landmark-based technique is not guaranteed to produce the same exact alignment on the same patient every time. However, once initial alignment is achieved, follow-up CTs could be easily registered to the initially aligned study. Nevertheless, our skull base alignment assessment did show excellent alignment of the chosen landmark (cochleas) with mean deviation of approximately 0.5 mm (1 voxel). Even more precise alignment could perhaps be achieved using more explicit segmentation of smaller structures (Liu et al., 2023; Zhang et al., 2024).

There was more variability in alignment of the axial plane along the AC-PC line than the coronal or sagittal planes. This is due to a less reliable anatomic relationship between the chosen landmarks and the brain structures of the anterior and posterior commissure. Again, if brain alignment in particular is desired, then a hybrid technique of registration with final cochlear alignment may be a better option.

There was also worse (moderate) interobserver agreement on AC-PC/sagittal plane measurement for our landmark registration method. One possible explanation for this is that our landmark method preserved all data along the z axis with rotation by adding slices, increasing the field-of-view cranio-caudal and causing a more zoomed out appearance on the sagittal images. This potentially introduced more variability into measurement. Also, the consistently 5° “off” nature of sagittal alignment with the landmark method could have introduced an element of bias as measurers discerned a pattern.

Only one CT scanner vendor (GE) was used for this study, though multiple scanner models were utilized. Performance of this CNN would require further validation and training on additional vendors.

Finally, this alignment method and test data are publicly available at https://github.com/radiplab/ct_head_align.

## 5 Conclusions

This study demonstrates an automated and simple landmark-based registration technique for precisely aligning skull base structures on head CTs while approximating AC-PC orientation. This has potential applications in both clinical and research settings, particularly for imaging focused on the skull base.

## CRediT authorship contribution statement

**Justin A. Cramer:** Writing – review & editing, Writing – original draft, Visualization, Validation, Supervision, Software, Resources, Project administration, Methodology, Investigation, Formal analysis, Data curation, Conceptualization. **Trevor Huff:** Writing – original draft, Validation, Software, Methodology, Investigation, Formal analysis, Data curation, Conceptualization. **Sean Kelly:** Writing – original draft, Validation, Methodology, Formal analysis. **Daniel Welch:** Writing – original draft, Validation. **Devin DeLuna:** Writing – original draft, Validation. **Conner Beyersdorf:** Writing – original draft, Validation. **Robin High:** Writing – original draft, Formal analysis. **Matthew White:** Writing – original draft, Conceptualization.

## Consent to participate

Consent was waived by the IRB for this retrospective study.

## Consent to publish

Consent was waived for publication given the absence of any patient-specific data in the publication.

## Prior presentations

None.

## Ethics approval

Approval was granted by the Institutional Review Board of the University of Nebraska Medical Center.

## Funding

The authors declare that no funds, grants, or other support were received during the preparation of this manuscript.

## Declaration of competing interest

The authors declare that they have no conflict of interest.

## Data Availability

I have shared my code and limited test data at https://github.com/radiplab/ct_head_align
